# Displacement Estimation Using 3D-Printed RFID Arrays for Structural Health Monitoring

**DOI:** 10.3390/s22228811

**Published:** 2022-11-15

**Authors:** Metin Pekgor, Reza Arablouei, Mostafa Nikzad, Syed Masood

**Affiliations:** 1 Department of Mechanical and Product Design Engineering, Swinburne University of Technology, Hawthorn, VIC 3122, Australia; 2 Data61, Commonwealth Scientific and Industrial Research Organisation, Pullenvale, QLD 4069, Australia

**Keywords:** RFID, 3D printing, structural health monitoring, direction of arrival, multiple signal classification

## Abstract

Radio frequency identification (RFID) tags are small, low-cost, wearable, and wireless sensors that can detect movement in structures, humans, or robots. In this paper, we use passive RFID tags for structural health monitoring by detecting displacements. We employ a novel process of using 3D printable embedded passive RFID tags within uniform linear arrays together with the multiple signal classification algorithm to estimate the direction of arrival using only the phase of the backscattered signals. We validate our proposed approach via data collected from real-world experiments using a unipolar RFID reader antenna and both narrowband and wideband measurements.

## 1. Introduction

Radio-frequency identification (RFID) relates to recognising physical items through radio communication [1]. RFID-based technologies are widely utilised as tracking sensors in supply chain management, transportation, logistics, and healthcare [2]. An RFID system basically involves an RFID reader, antenna, and RFID tags. These tags are classified based on their power source as active or passive or based on their operational frequency bands as low frequency (LF), high frequency (HF), very high frequency (VHF), ultra-high frequency (UHF), and super high frequency (SHF). With these features, RFID readers and tags can be connected to wireless sensor networks (WSNs) as sensor nodes.

Within the scope of the Internet of things (IoT) and structural health monitoring (SHM), RFID tags have been used as damage sensors [3]. Temperature, corrosion, and humidity can be measured and logged using RFID sensors embedded in structures [3,4,5]. Structures such as buildings, railways, dams, bridges, towers, and advanced machinery in industrial applications, on the other hand, can be independently monitored and intelligently controlled for physical, mechanical, chemical, and electrical properties by deploying electronic damage sensors. Damage sensors play an essential role in early damage detection in structural health monitoring (SHM). They increase safety, extend durability, and reduce maintenance costs and time. Various sensors, such as piezoelectric, fibre optic (FO), and magnetostrictive, are frequently utilised in SHM [6]. While FOs are usually used on the surface of existing structures or embedded in new structures, self-sensing sensors are mixed into the composite structure during manufacturing [7]. Thus, embedded sensor applications in SHM may vary according to material (composite or metal) and structure. While non-embedded sensors such as proximity sensors and visual cameras [8] may need additional wire connections and have location/installation issues on the structure, especially for real-time monitoring, however; embedded battery-less RFID-based sensors are more advantageous in SHM applications for being passive, wireless, and cost-effective [9].

There are two primary options for developing passive RFID-based SHM systems. The first option is to use existing RFID tags available on the market. The second option is to design and develop new RFID tags, antennas, or sensors that are suitable for any given SHM application. The second option clearly poses more challenges compared to the first one [3]. In general, devising and constructing new RFID tags or sensors can yield more precise solutions. However, utilising off-the-shelf RFID tags provides significant cost and time savings in SHM.

SHM techniques involve sensing, data processing, evaluation, and systems integration. In general, damage can be described as a variation that adversely affects a system’s existing or future performance [10]. Some damages impact the component level and do not affect the whole system’s performance, such as minor defects or flaws. However, minor damages such as corrosion and fatigue may escalate over time and affect the whole structure, resulting in catastrophic failures at the material level in structural and mechanical systems. This includes, for instance, monitoring the inclination angle of slender structures such as high buildings, towers, and chimneys, which have a high height-to-base area ratio. Leaning instability could occur if the overturning moment generated by a slight increase in inclination is equal to or greater than the resisting moment caused by the foundation. More than 5% of the inclined structures are usually demolished [11]. In such structures, it is necessary to periodically monitor the incline of the building to prevent possible catastrophic accidents. Therefore, in SHM applications, having information on a structure’s displacement and direction is quite significant [12]. Damage parameters for local displacements, such as crack location, length, and growth rate, and for global displacement, parameters such as deflections are critical for monitoring and determining the structure’s overall condition [13].

Slow movement or displacement, excluding the structure’s natural vibration and periodic movements, can result from structural damage. Displacements, tilts, curvatures, and strains are generally utilised to identify damage in static testing [14]. To detect displacement or vibration in situ, traditional technologies use contact sensors; however, these conventional systems can be costly. Most non-destructive SHM techniques include visual inspections that can only detect noticeable damage on the surface [15]. The RFID tags can be utilised as a sensor in indoor and outdoor applications on the structure without requiring any additional sensor or device, allowing the structures’ displacement to be detected remotely. In addition, they can be attached to the structures externally using protective materials resistant to high temperatures or humidity levels and are suitable for encapsulation techniques through emerging fabrication methods such as additive manufacturing (AM).

Encapsulation is essential in SHM applications to protect sensors from any chemical, mechanical, electrical, or physical detrimental impacts on the environment. RFID sensors are encased to protect them from harsh internal or external environments via moulding, potting, underfilling, glob top, or by more recent AM processes, also known as 3D printing [16]. 3D printing is useful for developing wearable and geometry-compatible sensors [17,18]. It is a fabrication method through which printed objects are built layer by layer using polymers, metals, or composites. Depending on the raw material used, 3D printing, or AM, may involve various fabrication methods such as sheet lamination, photo-polymerisation, binder jetting, material extrusion, powder bed fusion, and directed energy deposition. For polymer 3D printing, fused deposition modelling (FDM) is a common, cost-effective method based on the material extrusion technique. Most recently, the fabrication of encapsulated wearable sensors is often carried out using the FDM method rather than the traditional moulding encapsulation techniques, given its versatility in producing complex geometries from a range of thermoplastic polymers. Thermoplastics are traditionally produced by moulding processes such as rotational moulding, injection moulding, blow moulding, compression moulding and extrusion moulding. The benefits of one over another rely primarily on end-product specifications. However, heating temperature, recrystallisation time and other parameters directly impact the final product. For instance, in compressing moulding method, which is the most commonly used in industry, problems such as flashing, under-curing, over-curing, over-loading or under-packet, too easy or too stiff material flow, and gas trapping may occur during the process [19]. These issues lead to errors in sensor array encapsulation, such as non-linearity and unequal array gaps. Another competing 3D printing process adopted for the production of embedded electronics is the direct-write (DW) process. However, its use is not widespread due to its high cost and limited printing dimensions, and it is primarily suitable for thermosetting polymers.

Some relevant algorithms have been developed in the literature to estimate motion and displacement. Localisation technologies involving trilateration, triangulation and fingerprinting are generally employed with RFID devices [20]. These methods frequently use passive RFID tags for position estimation and damage detection. In trilateration, which is a range-based location estimation method, position estimation mainly relies on the knowledge of the location of the existing reference tags. The RFID indoor positioning systems utilise Bayesian, Gaussian-mixture-based, and *k*-nearest neighbour algorithms [21]. These algorithms yield centimetre-level accuracies. However, they require many tags and RFID antennas. Moreover, trilateration-based positioning using the received signal strength indicator (RSSI) readings is challenging in indoor applications due to high levels of noise, interference, and multipath propagation [22]. In addition, in distance-dependent multipath modelling, an RFID microchip’s input impedance is power dependent making RSSI calibration complex [23]. Due to these reasons, the trilateration method has mainly been considered ineffective in the related scientific literature [24]. The triangulation technique is another range-based location estimation method, which is phase-based, that exploits the information in the received signal phases for position estimation. The phase of the received signal can depend on frequency or power [21]. In contrast to distance measurements, these signals can be calibrated in phase difference of arrival applications [23]. Therefore, triangulation (phase-based) methods are more reliable, accurate, and robust compared to trilateration (distance-based) methods. Advanced RFID reader devices can measure signal phases with high accuracy. For example, they can measure the incoming signal phase with an accuracy of approximately 0.27°, potentially leading to millimetre-level accuracy in the estimation of position and, consequently, motion [25]. The fingerprinting technique is a range-free location estimation method, with the benefits of scene features from the surrounding signatures with RSSI and phase at each location in the areas of interest to build a fingerprint database [26]. As a result, this method is useful for mitigating the effects of NLOS and multipath in challenging indoor environments [20]. The fingerprint location estimation accuracy can be further increased with machine learning algorithms [27]. Aside from these techniques, image processing-related techniques, including RF hologram [28] and tomography [29], give more accurate results(mm-levels) with phase data for moving tags tracking.

Whether range-based or range-free, the displacement can be estimated using RFID sensors in conjunction with location estimation techniques. Changes in RFID tags’ positions due to changes in the structure’s geometry to which they are attached can be monitored wirelessly through variations in the received signal strength indicator (RSSI) or received signal phase [30]. The changes may involve azimuth or elevation angles. In the literature, RFID tags have been used as displacement (motion) detection sensors on constructions, people, or vehicles [25,31,32,33,34,35,36,37]. While RSSI is mainly preferred for outdoor environments and mobile applications, for indoor applications and stationary situations, using phase data is more advantageous in terms of noise figures in the measurements [38]. In addition, in these applications, the radar cross-section (RCS) can be estimated by RSSI and is used to enhance the radiation distance [39]. However, when used with multiple tags and receiver antennas, multiple operating frequencies, a synthetic-aperture radar (SAR) antenna, or accelerometer sensors, the phase-based RFID location estimation techniques are more accurate (millimetre level) than RSSI-based methods [25]. As a result, displacement estimation accuracy changes depend on the measurement method, environment, and test parameters such as frequency, sensor number, antenna number, and measurement distance.

The motivation of this article is to develop an idea for the more widespread use of passive RFID tags as damage sensors in structures for SHM systems without the need for any other embedded sensors. To this end, a study is presented here to give insight into how to use off-the-shelf passive RFID tags as sensors encapsulated by a material extrusion-based3D printing method, also known as Fused Filament Fabrication (FFF), in the structures. It has been investigated together with the direction of arrival (DOA) and the multiple signal classification (MUSIC) techniques so that passive tags can detect the displacements. First, we encapsulate UHF passive RFID tags with polymer using a FFF 3D printing process so that arrays of them can be installed on the structure surfaces. Using a monostatic RFID reader equipped with only a single polarisation antenna, we measure and calculate only the backscattering signal phases and process them using the MUSIC algorithm to estimate the DOA for the associated motion. These include rotating, twisting, stretching, and bending movements, which can cause damage to the structure. We experiment with line, matrix, flexible line, and twistable line arrays, as well as various RF bands. We collect measurements indoors or in an anechoic chamber environment. Our findings demonstrate that 3D-printed RFID tag arrays can be utilised as (angular) displacement sensors to monitor building structure movements in a non-invasive and cost-effective fashion.

Our major contribution in this paper is around the effective utilisation of 3D-printed RFID tags and the MUSIC algorithm to estimate angular displacement for SHM applications. The merits of this approach are also evident in a few existing related works, such as [23,35], where RFID tags are embedded in textiles for low-cost detection of coarse motion. Although our work leverages a similar premise, it opens new avenues for scalability and wider uptake of such approaches in SHM applications. This is mainly thanks to far more effective encapsulation capabilities afforded by employing 3D printing (AM) technologies.

## 2. Materials and Methods

In this section, we present the details of the utilised 3D encapsulation technique with AM, Signal phases in RFID-based systems, signal modelling, assumptions, and DOA estimation using the MUSIC algorithm.

### 2.1. RFID Tag Encapsulation via AM

In additive manufacturing (AM), encapsulation technologies are used to produce sensors [40], actuators [41], micro-electromechanical systems [42], batteries, memory alloys, reinforcing fibres [43], and optical fibres [44]. The AM process, as opposed to the conventional subtractive manufacturing methods, enables manufacturing in the entire build volume using a layer-by-layer deposition. Therefore, AM can create cavities and features where components or sensors can be embedded during or after printing.

In this study, we use the polypropylene (PP) polymer as the 3D printable encapsulation material. PP has good fatigue and chemical resistance properties. It also has high impact and flexural strength due to its semi-crystalline properties. In addition, PP has a low dielectric loss factor [45], making it nearly transparent in a broad range of RF frequencies. Therefore, it is suitable for RFID encapsulation applications.

In the 3D-printing encapsulation process, we first design a cavity using a computer-aided design/manufacturing (CAD/CAM) software called Creo Parametric developed by PTC, Boston, MA, USA and print it via AM to embed an external component. Then, we pause the 3D printing process to embed the external component. Afterwards, we resume the printing process, if required and finally, we encapsulate the desired component through the AM process [46]. 

We adopt fused filament fabrication (FFF) 3D printing technology in this work. In FFF printing, all the above steps can be controlled manually to build an object layer by layer via extruding thermoplastic materials such as ABS and PP. However, manual interventions can cause the formation of the substrate and pattern design to adjust during the process, which is of great importance to the behaviour of polymers within electromagnetic fields, particularly regarding refraction and scattering. 

We use off-the-shelf Alien ALN-9762 Short Inlay RFID tags as passive sensors. Their operating frequencies are 840 MHz to 960 MHz, and they have a 32 bits tag ID (TID) for authentication and 128 bits electronic pin code (EPC) of user memory for distributed data applications. In addition, the tags have their meander-type antenna with dimensions of 70 mm by 17 mm.

Using the 3D CAD program, we draw the PP substratum in two parts. The first part has a thickness of 1.5 mm and is printed by an Ultimaker ^3^ FFF 3D printer manufactured by Ultimaker, Utrecth, The Netherlands. The printer can fabricate geometries using various filaments, such as Nylon, ABS, PLA, CPE, and water-soluble PVA, and has a filament diameter of 2.85 mm and a printing resolution of 20 microns. The extruder and bed temperature were adjusted during PP printing to 230 °C and 90 °C, respectively, to ensure suitable viscosity and flow rate. The 3D printing parameters for PP are shown in Table 1.

**Figure 1:**
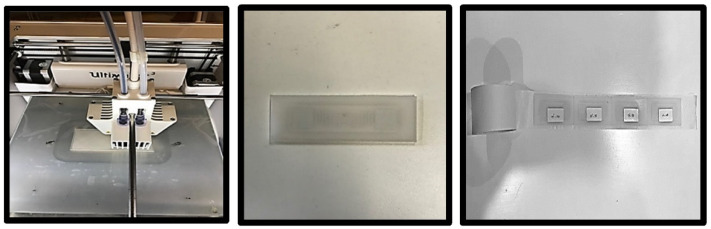
RFID sensor encapsulation using a 3D printer and arranging them into a sensor array.

Then, we place the RFID tags on the substrate before printing the upper PP substratum, which is 1.4 mm thick. Finally, to create ULA sensor series, we position the encapsulated RFID tags equidistantly to arrange them into a linear array on the adhesive tape (see Figure 1).

### 2.2. Signal Phases in RFID-Based Systems

RFID systems use two-way communication. An RFID reader consists of a local oscillator, transmitter, receiver, antenna, and circulator and communicates with passive tags via backscattering signals. The RFID readers are generally characterised by their range, i.e., the maximum distance to which they can be identified. As in other RF systems, the transmitted signal amplitude, frequency, and system phase response impact the performance of the RFID systems. The signal transmission is affected by thermal noise and phase noise, which should be minimised to achieve high precision. The thermal noise depends on the temperature and bandwidth; thus, using a narrow frequency band reduces the receiver’s thermal noise.

The phase noise is caused by the circulator, which is a three-port device where the RF signal travels from the antenna to the receiver port or from the transmitter to the antenna port. In a circulator, the transmitter port and receiver ports are isolated. However, in a nonideal circulator, leakage can occur between the ports. This leakage leads to phase noise, which is more troublesome compared to thermal noise.

**Figure 2:**
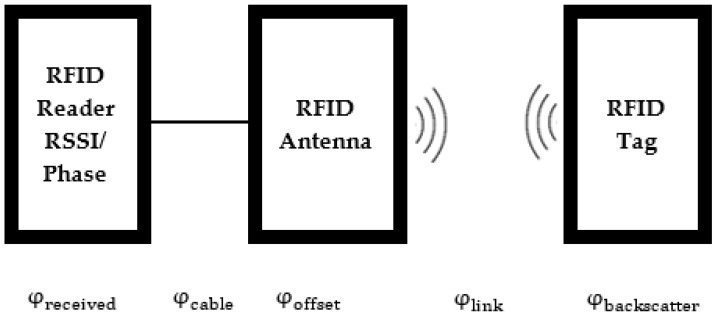
The signal phases in RFID systems.

The free space path loss is a significant attenuator of the RF signal. Multipath propagation can also result in significant signal loss. In a multipath environment, RF signals reach the receiver via two or more paths. These signals can superimpose destructively due to different phase delays. To minimise the adverse effects of multipath propagation, several diversity techniques, such as time, angle, frequency, space, and polarisation diversity, have been developed [47]. We use a monostatic RFID reader (ST25RU3993-EVAL produced by STMicroelectronics, Geneva, Switzerland) with a single-polarisation antenna in this work. It contains a directional coupler and self-jamming cancellation circuit to mitigate the phase noise. Self-jamming signals are due to reflections from close objects or transmit-receive antenna coupling. The carrier cancellation circuit reflects a certain amount of the coupled power into the coupling port, combined with the self-jamming signal at the directional coupler’s isolated port. The leakage phase noise is minimised if the carrier and reflected signals have opposite phases. Backscattering signals are modulated in the most recent RFID tags using a binary phase-shift keying scheme. As a result, the local oscillator signal and the transmitted signal are not mixed at the receiver of the RFID reader. Thus, the transmitter and receiver signal phases do not affect each other. A typical RFID system’s signal phases associated with different communication stages are shown in Figure 2.

As per Figure 2, we have

(1) $$\varphi_{\mathrm{received}}=\varphi_{\mathrm{cable}}+\varphi_{\mathrm{offset}}+\varphi_{\mathrm{link}}+\varphi_{\mathrm{backscatter}}$$

where

$\varphi_{\mathrm{received}\;}$ is the tag signal phase;
$\varphi_{\mathrm{cable}}\;$ is the phase change due to the coaxial cable;
$\varphi_{\mathrm{offset}}$ is the phase change due to the antenna and other components, such as the divider and combiner;
$\varphi_{\mathrm{link}}$ is the phase change due to the propagation in free space; and
$\varphi_{\mathrm{backscatter}}\;$ is the backscatter signal phase.

The phase change due to the coaxial cable, a non-dispersive device, is linear. It depends on the cable’s physical length and the material’s dielectric constant that fills the cable and insulates the core from the shell. The cable phase is given by

(2) $$\varphi_{\mathrm{cable}}=\frac{L\sqrt{\epsilon }}{c}$$

where

*L* is the cable length (m);
*ϵ* is the dielectric constant of the insulator; and
*c* is the speed of light ($2.9979\times 10^{8}$ m/s in the air).

Consider the distance between the RFID reader antenna and the tag to be *D.* Thus, the propagation distance is twice the physical distance (2*D*), and the propagation phase is

(3) $$\varphi_{\mathrm{link}}=\frac{2D}{\lambda }\;\mathrm{mod}\;2\pi$$

in the RFID reader ST25RU3993, the two input mixers are controlled by LO signals with a 90° phase shift to construct an IQ demodulation circuit. Thus, the circuit provides the in-phase (*I*) and quadrature (*Q*) component values of the received signal. The phase of the backscattering signal sensed by the RFID reader is calculated as [30]

(4) $$\varphi_{\mathrm{received}}=\tan^{-1}(Q/I)\;(-\pi ,+\pi )$$

Finally, in discrete time measurements, if the previous measurement will be a reference to the subsequent measurement, the factors affecting the signal phase associated with different communication stages, such as cable length, should be considered.

### 2.3. Signal Model

**Figure 3:**
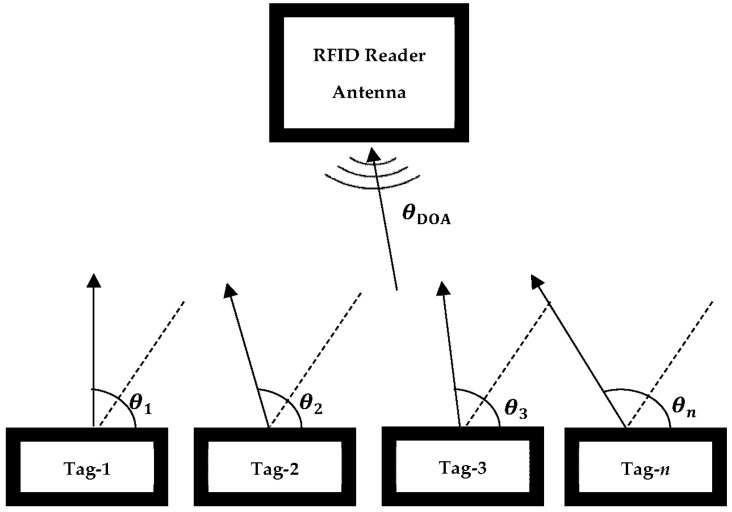
Modelling of signals backscattering from passive RFID tag series.

The transmission of backscattering signals from the passive tags to the reader is shown in Figure 3. Considering an RFID system is composed of an RFID reader, a reader antenna, and a sensor array (tag array), the received signal from the RFID reader can be determined as

(5) $$T(t)=A_{t}\left(\theta \right)S_{t}\left(t\right)+A_{i}\left(\theta \right)S_{i}\left(t\right)+n(t)$$

where $S_{t}\left(t\right)$ and $S_{i}\left(t\right)$ denote the RFID reader’s interrogation signal (transmitted signal) and the backscattering signal (incidence signal) from the passive UHF tag, respectively. Similarly, $A_{t}\left(\theta \right)$ and $A_{i}\left(\theta \right)$ are the Vandermonde matrixes, which show the signals from the reader’s and the sensor’s steering vectors, and *n*(*t*) is an additive white Gaussian noise (AWGN) vector.

We assume that the signal transmitted from the monostatic RFID reader is only for sensor excitation. In this case, when sensors are spaced uniformly on a line to form a uniform linear array (ULA), the total signal received at the RFID reader antenna $T\left(t\right)$ includes only the backscattering signal $S_{i}\left(t\right)$ and the noise $n\left(t\right)$. At the time domain, it can be shown as

(6) $$T\left(t\right)=A_{i}\left(\theta \right)\;S_{i}\left(t\right)+n\left(t\right)$$

where

$A_{i}\left(\theta \right)$ is a steering vector;
$S_{i}\left(t\right)$ is a backscattering signal vector;
*n(t)* is the additive white Gaussian noise;
θ refers to the position angle of the sensor;
*i* is the number of transmitter sources.

### 2.4. Main Assumptions

We make some commonly accepted assumptions. First, all RFID tags are identical and have the same radiation pattern. Second, all the backscattering signals the RFID reader receives have the same frequency and travel over the same distance. Third, all equally spaced and flat RFID tags lie on the same plane. Fourth, the RFID reader antenna is linear, single-polarised, and positioned in front of the tag array. Finally, the position of the tag array is fixed during the data collection process.

In applications for adjacent sensors, sensor spacing should not be more than *λ*/4 due to cos∅ ≤ 1 [25]. Therefore, linear array geometry must be designed before embedding, including moulding and 3D printing. Due to this restriction, adjustment of sensor location in the FFF 3D printing process is easier compared with conventional moulding process encapsulation, such as compression moulding.

**Figure 4:**
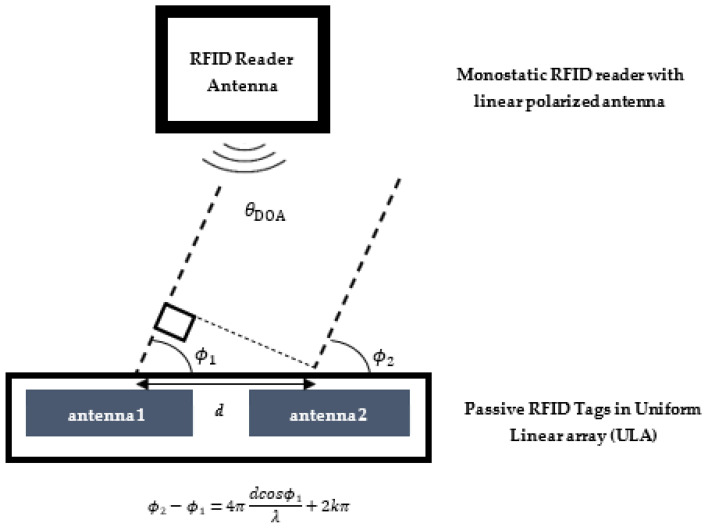
DOA in the spatial domain for two tags and a monostatic antenna.

For ULAs, sensor place is essential. For instance, as seen in Figure 4, for linear tags arrays, the phase between two antennas (passive tags) are

(7) $$\cos\emptyset =\lambda \frac{\left(\phi_{2}-\phi_{1}\right)}{4\pi d}$$

where

$\phi_{1}$ is the 1st sensor phase;
$\phi_{2}$ is the 2nd sensor phase;
λ is the wavelength; and
d is sensor spacing;
∅ is the observed phase between two tags’ backscattering signals.

In addition, the 3D printing material is not conductive and is not subjected to any stress that would cause deformation. The ground surface is also non-conductive due to the Fresnel zone. We keep the RFID receiver amplifier gain and transmitter attenuation constant. The additive Gaussian noise is uncorrelated with the received signal. The RFID signals are plane waves in far-field propagation. Far-field propagation occurs when the communication distance is larger than the Fraunhofer distance [31], defined as

(8) $$R_{F}=\left(2\delta^{2}\right)/\lambda$$

where δ is the largest dimension of the antenna. For instance, with f=921 MHz and a four-element array of typical RFID tags having meander-type 7 cm antennas, we have $R_{F}=49$ cm.

### 2.5. DOA Estimation

DOA estimation methods are frequently used in wireless applications such as radar, radio astronomy, navigation, and sonar. Recently, they have been used with RFID systems [48]. The angular location information, i.e., azimuth and elevation angles, can be determined using DOA estimation methods with RFID readers and tags.

DOA estimation methods retrieve the direction information of several electromagnetic waves/sources from the outputs of many receiving antennas that form a sensor array [49]. Several methods are available for estimating the DOA of the radio signals on the antenna array. DOA estimation approaches can be divided into three categories: traditional, subspace-dependent, and maximum-likelihood-based. The traditional methods rely on null steering and beamforming to improve estimation resolution. They typically require arrays with large numbers of elements. These methods are relatively straightforward in computation. Maximum likelihood and subspace-dependent methods are parametric with higher accuracy and resolution but incur higher computational complexity. Some parameters, such as backscattering RF signal strength, bandwidth, noise power, and array geometry, impact the performance of the DOA estimation methods [50].

Two popular DOA estimation algorithms are ESPRIT, the abbreviation for “estimation of signal parameters via rotational invariance techniques”, and MUSIC, an acronym for “multiple signal classification”. The MUSIC algorithm has high resolution and good estimation accuracy. It uses a peak search method to estimate the DOA. MUSIC is a subspace-based method that utilises a pseudo-spectrum via estimating the noise subspace from the signal autocorrelation matrix [51].

The measured signal autocorrelation matrix is defined as

(9) $$R_{x}=E[ΧX^{H}]$$

where X is the signal matrix containing all the measurements (snapshots), H denotes the Hermitian operator, and E represents the expectation operation. The noiseless signal autocorrelation matrix is represented by

(10) $$R_{s}=E\left[ss^{H}\right]$$

and the noise autocorrelation matrix by

(11) $$R_{n}=\sigma^{2}Ι$$

where Ι is the identity matrix of appropriate size. Therefore, we have

(12) $$R_{x}=AR_{s}A^{H}+R_{n}$$

Let $\lambda_{1}\geq \;\lambda_{2\;}\geq \ldots \;\geq \;\lambda_{M}$ ≥0 denotes the eigenvalues of $R_{x}$. Assuming K smallest eigenvalues correspond to the noise subspace and the rest to the signal subspace; the MUSIC algorithm uses the eigenvectors associated with the noise subspace and arranged within the matrix ${Ε}_{n}$ to define the MUSIC pseudo-spectrum as

(13) $$P_{music\;}\left(\theta \right)=\frac{1}{a^{H}\left(\theta \right){Ε}_{n}{Ε}_{n\;}^{H}a\left(\theta \right)}$$

where $a\left(\theta \right)$ is the steering vector parametrised by the angle θ that represents the angle of the signal incident upon the sensor array, i.e., the DOA. It is estimated by maximising the pseudo-spectrum $P_{music\;}\left(\theta \right)$.

**Figure 5:**
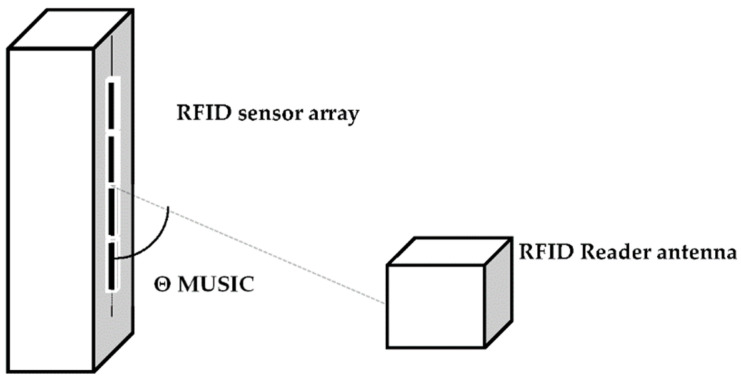
DOA estimation setup using the MUSIC algorithm.

The setup for backscattering signal DOA estimation using the MUSIC algorithm with a 4-element passive RFID sensor array is shown in Figure 5.

## 3. Experiments and Results

Using the MUSIC algorithm, we consider four test cases for detecting rotation, twisting, stretching, and bending movements by estimating the DOA for elevation and azimuth angle. Therefore, we utilise single-line, two-dimensional matrix, single flexible line, and twist line arrays. We use wideband (902.750–927.250 MHz) and narrowband (915 MHz, 921 MHz, and 928 MHz) measurements. Phase angle measurements were made separately for each physical displacement, so each previous measurement result references the next test. More than 700 data were collected for each test step. We make all tests indoors or inside an anechoic chamber.

### 3.1. Line Array

**Figure 6:**
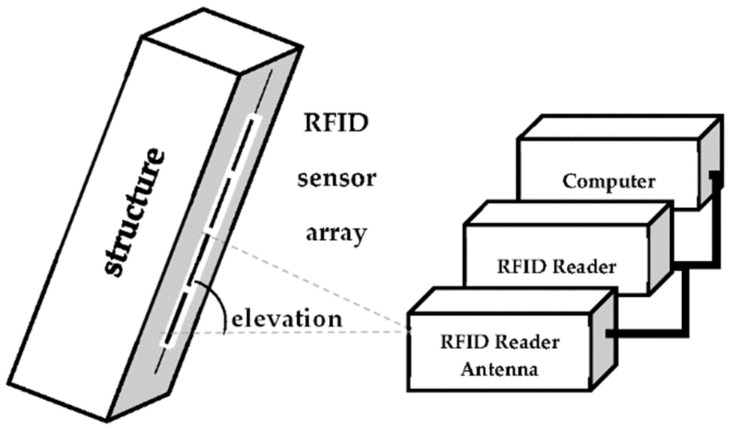
Test platform for motion detection.

Here, we estimate a tagged object’s position variation (displacement) using an RFID system and the MUSIC algorithm in an indoor environment. We use an RFID tag array installed on a wooden column. In the tests, the wooden block was used to prevent the tag from being negatively affected by metal and conductive surfaces in the near radiation field. The RFID reader antenna, which is single-polarised with a 6 dBi directivity gain, is positioned *λ*/2, *λ*, and 2*λ* away from the sensor arrays (see Figure 6). 

**Figure 7:**
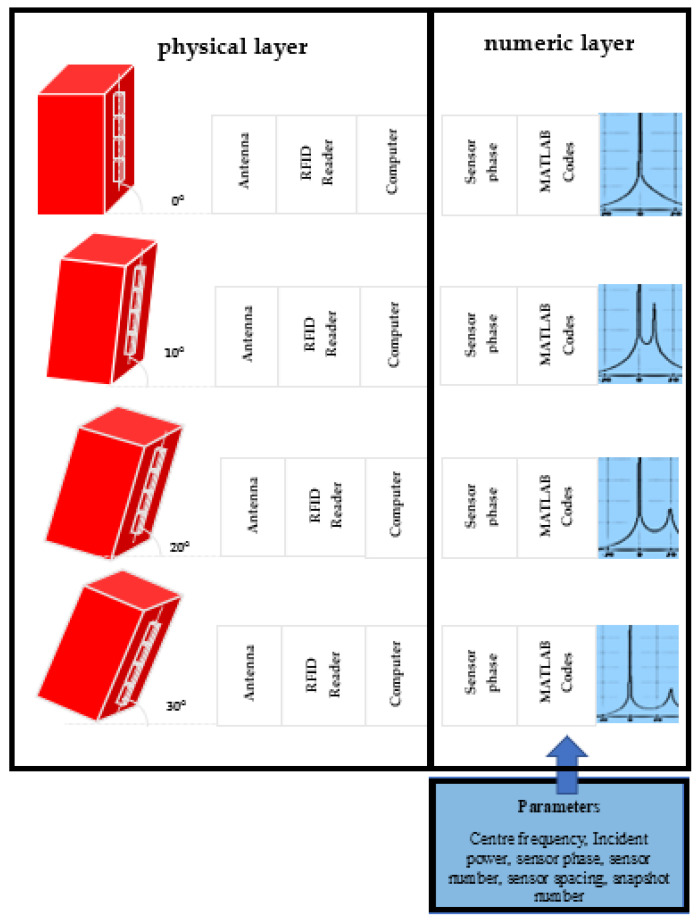
DOA estimation using the MUSIC algorithm with RFID tags for SHM.

Using the RFID reader, we collect all sensor backscattering data, including signal phase, for different elevation angles on the array. Then, we process the data and estimate the elevation angle through the MUSIC algorithm implemented using MATLAB software. Finally, we estimate the object’s position variation using the estimated angles and their changes over time (see Figure 7).

We conducted the experiment indoors and placed the RFID reader antenna and the sensor array on the floor. It leads to multipath propagation and hence power-dependent signal phase changes.

For the *λ*/2 distance, there is a linear increase in the estimation error as the elevation angle increases. In contrast, for the *λ* distance, the estimation error decreases as the elevation angle increases, mainly due to multipath propagation. However, for the 2*λ* distance, the estimation accuracy is relatively high where the DOA estimation error is around 1° for elevation angles between 0° to 30°.

### 3.2. Two-Dimensional Array

**Figure 8:**
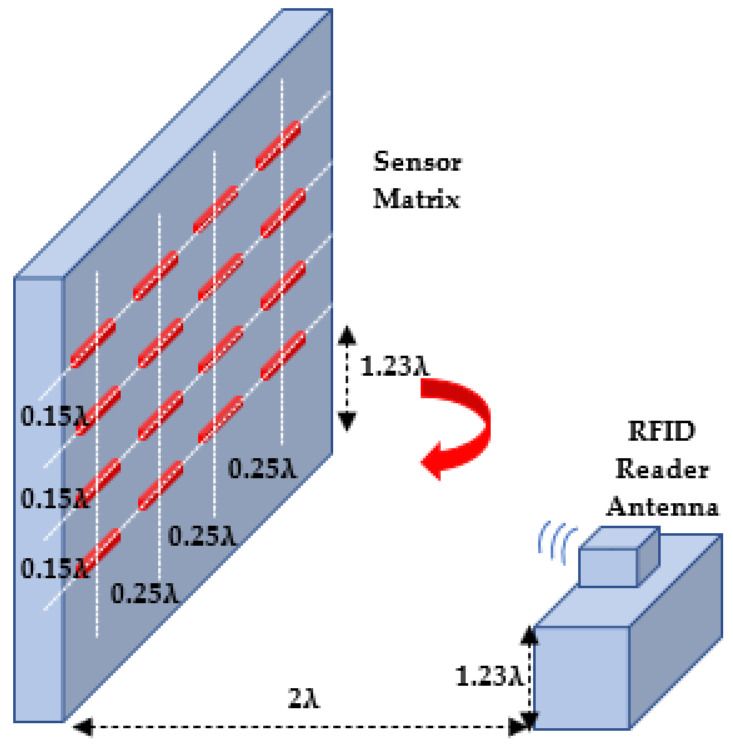
2D RFID tag array setup for DOA measurements.

**Figure 9:**
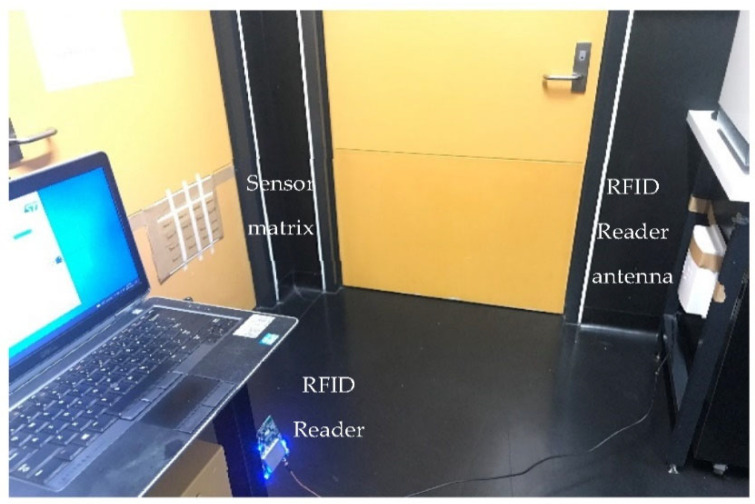
2D RFID tag array indoor applications test platform for DOA measurements.

In this experiment, we use 16 identical RFID tags. We arrange the tags in a two-dimensional matrix array (Figure 8 and Figure 9). Four rows are named A, B, C and D. Each has four sensors enumerated as 1, 2, 3, and 4 from top to bottom. They are installed on a wooden surface perpendicular to the floor. The distance between the rows is 0.15λ, and the distance between two tags in each row is 0.25*λ*. The RFID reader is situated 2λ away from the centre of the 2D sensor array and 1.23*λ* above the ground.

**Figure 10:**
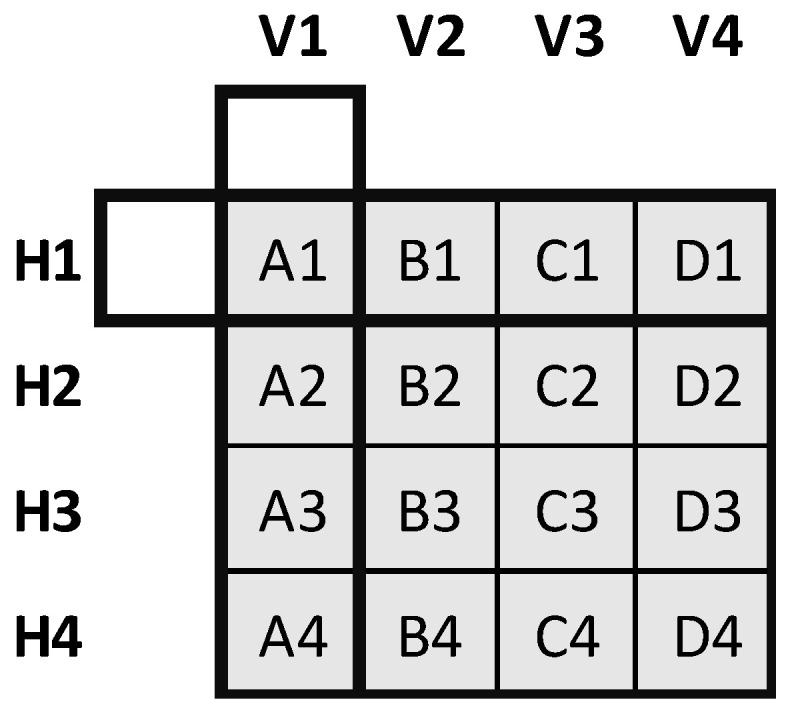
Utilised 2D sensor array. V1,…, V4 are the DOA of the columns and H1,…, H4 are the DOA of the rows estimated by the MUSIC algorithm.

We collect the signal IQ data when the azimuth angle of the array is 0°, 10°, and 20° using the frequency bands of 915 MHz, 921 MHz, and 928 MHz. We use the signal phase data to calculate the associated MUSIC spatial spectrum for each sensor row in both directions. Then, we estimate the angle corresponding to each row and column by maximising its associated MUSIC spectrum (see Figure 10).

For a greater number of sensor arrays, to increase the DOA performance, cascade algorithms such as MUSIC have already been used in the literature [52]. To this end, we calculated the MUSIC angle separately for each vertical and horizontal position at 0°, 10°, and 20° azimuth angles at 921 MHz operating frequency. Then, we used the estimated vertical DOA values (V1, V2, V3 and V4) in the MUSIC algorithm as a cascade again. The first MUSIC angle estimation was based on the inter-sensor distance of 0.15*λ*. Similarly, we used the estimated horizontal DOA values (H1, H2, H3 and H4) in the MUSIC algorithm again. The second MUSIC angle estimation was based on the inter-sensor distance of 0.25*λ*. The largest errors were 11° and 15° for the vertical and horizontal directions.

For the second test, we rotated the RFID reader antenna by 90°. The DOA estimation error is affected by the RFID reader antenna polarisation, sensor number and spacing, multipath propagation, and reading noise/error.

Although increasing the number of sensors and using the cascade MUSIC algorithm in matrix sensor tests increases the DOA estimation accuracy, it also causes data loss due to increased distance. For this reason, RFID reader output power, antenna gain, and radiation pattern should be considered to minimise data loss.

A 2D sensor array can help determine twisting and stretching caused by asymmetric stress on the structure. For instance, an RFID reader antenna can be placed at the rigidity centre in a tall building. Moreover, a 2D sensor array installed on a shear wall can help monitor the stiffness of the shear wall when it is exposed to any external stress, such as lateral load due to an earthquake.

### 3.3. Flexible Line Array

**Figure 11:**
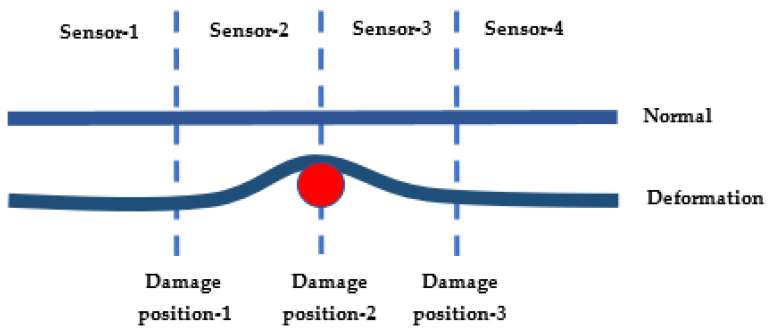
Test setup for bending detection.

**Figure 12:**
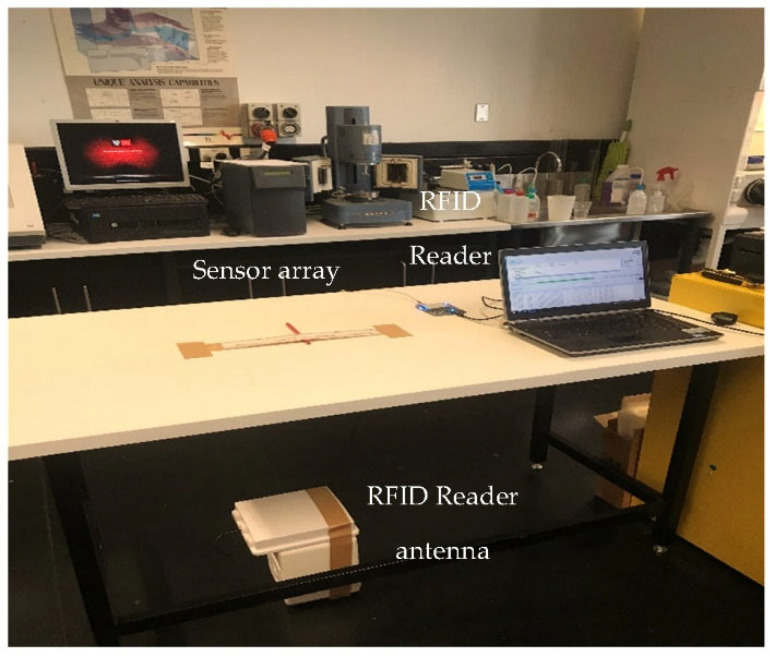
Test platform for bending detection.

We conducted this experiment using four identical RFID tags on the test platform 0.15*λ* apart (see Figure 11 and Figure 12). We placed the RFID reader 2*λ* away from the array. After preparing the test platform, we emulated artificial damage at three different locations below the sensor array by placing a round wooden object with a 3 mm diameter.

We collected data and processed it via the MUSIC algorithm to find the damage locations. The presence of the damage can be detected via the MUSIC algorithm, but not the location of the bending damage. Therefore, in addition to DOA estimation using the MUSIC algorithm, extra information is required for position estimation, including the phase changes of the array elements.

We compare the DOA estimated via the MUSIC algorithm for the normal state with that of the state with possible damage. Any difference indicates the existence of damage. We then check the maximum phase deviations between the sensors to determine the damage location. This way, we find the position of the bend.

### 3.4. Twist Line Array

**Figure 13:**
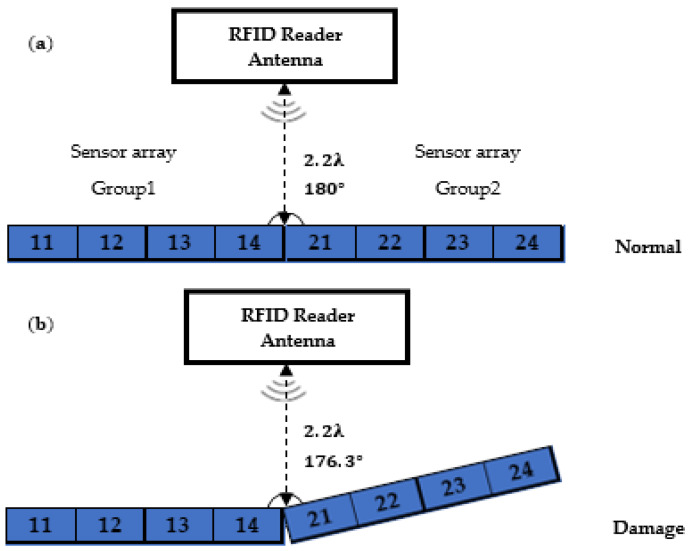
Twist line array test setup, (**a**) normal conditions, (**b**) damaged.

**Figure 14:**
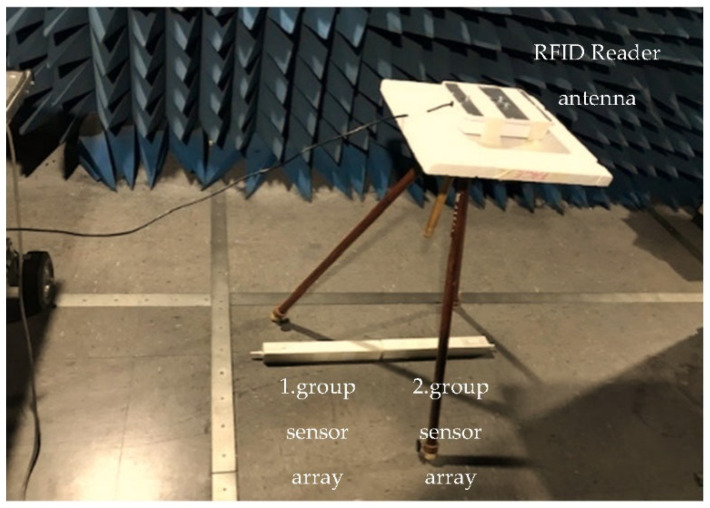
Twist line array test platform in an anechoic chamber.

In this experiment, we use eight identical 3D-printed RFID sensors encapsulated by PP with 2.5 cm thickness and installed on two woodblocks, each 5 cm wide, 5 cm high, and 40 cm long (see Figure 13 and Figure 14), with sensor spacing of 0.08*λ*.

We place the sensor arrays in two groups on the floor wooden blocks. In the first group, sensors are labelled as 11, 12, 13 and 14 and sensors are labelled as 21, 22, 23 and 24 in the second group. We place the RFID reader antenna linear and single-polarised with a 6dBi gain, 2.2*λ*, away from the sensor arrays (see Figure 13 and Figure 14). The two sensor arrays are attached to one end to make a straight line. Using a board, we simulate structural damage by lifting the sensor array on the right by 3.87°.

We consider three different test procedures to estimate the DOA and accordingly detect the damage. In the first procedure, we use the entire RFID frequency band (915–928 MHz with 0.25 MHz hopper frequency). We then estimate the DOA using the MUSIC algorithm. The DOA estimation error was 2.21°. In the second procedure, we repeat all measurements using 915 MHz, 921 MHz, and 928 MHz frequency bands. We then estimate the corresponding DOAs for each frequency band and compare them with the ground-truth value. The DOA estimation errors were 3.21°, 0.79°, and 1.21°, respectively, with an average value of 1.73°. In the third test procedure, we consider each sensor group independently and estimate the DOA values using wide and narrow frequency bands as in the first and second procedures. By using the wideband measurement in the initial condition, DOAs of the first and second sensor groups are estimated as 49° and 41°, respectively. For the damage state, DOAs are estimated as 36° and 51°, respectively. Similarly, for narrowband measurements, at 915 MHz, DOA estimates are 17° and 25° for the original and damage states, respectively. At 921 MHz, for the initial and damage conditions, DOAs are 18° and 11°, respectively. Finally, at 928 MHz, for the original and damage states, DOAs are 17° and 13°, respectively. Therefore, on average, the DOA estimation error in these tests with narrowband measurements was on average, 2.26°. The test results show that using a wide frequency band improves the accuracy of each test procedure. In addition, averaging the results for multiple narrow frequency bands enhances accuracy.

## 4. Summary of Results

We summarise the results of the tests with different sensor numbers and bandwidths and consider damage scenarios in Table 2. Overall, the results of fewer sensors and narrowband measurements were more promising compared to higher numbers of sensors and wideband measurements. We also list the parameters affecting the test results in Table 3. Bandwidth, sensor number, and environment are the main factors, according to empirical research. Although increasing the number of sensors improves the accuracy in some scenarios, it also results in loss of signal and data.

In the literature, there are several works that use RFID sensor arrays to estimate displacement via various methods. In a relevant study, a displacement estimation accuracy of 8.67 cm is achieved using RSSI and RCS measurements associated with RFID tags [53]. Another study, which is closely related to our work in methodology, adopts a phase-based approach to estimate angular displacements using RFID tag arrays [23]. However, the authors of [23] utilise near-field communication and embed RFID tags inside textiles without employing any advanced encapsulation technologies such as 3D printing. They achieve accuracies of about 8–12 degrees or errors of about 4–7%, while, in our work presented in this paper, we achieve accuracies of around 1 degree or errors of less than 3% that correspond to millimetre-level displacement estimation accuracies. Moreover, the results of [23] are obtained using two sensor arrays while we use a single array in similar settings, albeit in a far-field signal regime and using 3D-printed sensors.

## 5. Conclusions

Off-the-shelf RFID tags with meander-type antennae are frequently used to identify objects and people. In this work, we have implemented a novel process of embedding the RFID tags into PP polymer via 3D printing using an FFF 3D printer. We have then explored using the manufactured embedded RFID tags as damage sensors for SHM. To this end, we used linear and matrix sensor arrays in conjunction with the MUSIC algorithm to estimate the DOA. Any change in the DOA can indicate movement and possible structural damage. Unlike other studies in the literature, we encapsulated the sensors in the 3D printing process, which is a cost-effective and scalable way the development of wearable and geometry-conforming sensor structures. We have made discrete-time measurements with narrowband and wideband frequencies, making more accurate estimates based on previous tests. We applied the cascade MUSIC algorithm to matrix passive sensors. We tested the applicability of passive tags as angular displacement, especially in indoor spaces. We considered potential damage scenarios associated with rotation, twisting, stretching, and bending movements, which are essential for monitoring the health of any structure. The results showed that 3D-printed RFID tag arrays could be utilised as angular displacement sensors with excellent accuracy to monitor the movements of building structures in a non-invasive and cost-effective fashion. In future studies, we plan to examine new measurement techniques to increase the sensing range, which is limited by the regulations and use machine learning algorithms for ULA and non-ULA passive sensor arrays to minimise the sensor number and improve the accuracy. In addition, we will explore the potential of addressing noise issues, which may hinder the realisation of our approach at a large scale, by fine-tuning the material encapsulation characteristics to achieve high-quality signals and datasets, which can, in turn, lead to obtaining models with much higher accuracies. We will also consider implementing and testing our system on real structures made from timber, metal, and concrete.

## Figures and Tables

**Table 1:** PP 3D printing parameters.

Printing Surface	Heating Bed, Glass Surface
Bed temperature (°C)	90
Printing temperature (°C)	230
Infill degree	60%
Infill orientation	Crossed (45°)
Printing speed (mm/s)	30
Layer thickness (mm)	0.2

**Table 2:** Summary of the results.

Experiment	DOA Estimation Error
elevation angle measurement with 4 sensors (line array)	1° error up to 30° elevation
elevation angle measurement with 8 sensors (line array)	1° error up to 18° elevation
azimuth angle measurement with 16 sensors (matrix array)	1° error up to 1.81° azimuth
elevation angle measurements with narrowband (line array)	1° error up to 18.42° elevation
elevation angle measurements with wideband (line array)	1° error up to 7.58° elevation

**Table 3:** Parameters affecting the test results.

Parameter	Variation	DOA Resolution with MUSIC
sensor number	increase	inconclusive
sensor surface area	increase	increase
snapshot number	increase	increase
sensor spacing	increase	decrease
sensor position	change of orientation	inconclusive
frequency	change of frequency	inconclusive
RFID reader output power	increase	inconclusive
frequency band	increase	increase
bandwidth	increase	inconclusive
RFID reader distance to the ground	increase	increase
RFID reader distance to the sensor array	increase	decrease
RFID reader polarisation	single or circular	inconclusive
sensor 3D printing thickness	increase	decrease
sensor 3D printing material	change of dielectric constant	inconclusive
noise	increase	decrease

## Data Availability

Data available upon request.
